# A systematic review of risk factors associated with depression and anxiety in cancer patients

**DOI:** 10.1371/journal.pone.0296892

**Published:** 2024-03-29

**Authors:** Deborah Ikhile, Elizabeth Ford, Devyn Glass, Georgie Gremesty, Harm van Marwijk

**Affiliations:** 1 Department of Primary Care and Public Health, Brighton and Sussex Medical School, University of Sussex, Brighton, United Kingdom; 2 National Institute for Health and Care Research Applied Research Collaboration Kent, Surrey and Sussex, Hove, United Kingdom; The Chinese University of Hong Kong, HONG KONG

## Abstract

Depression and anxiety are common comorbid conditions associated with cancer, however the risk factors responsible for the onset of depression and anxiety in cancer patients are not fully understood. Also, there is little clarity on how these factors may vary across the cancer phases: diagnosis, treatment and depression. We aimed to systematically understand and synthesise the risk factors associated with depression and anxiety during cancer diagnosis, treatment and survivorship. We focused our review on primary and community settings as these are likely settings where longer term cancer care is provided. We conducted a systematic search on PubMed, PsychInfo, Scopus, and EThOS following the PRISMA guidelines. We included cross-sectional and longitudinal studies which assessed the risk factors for depression and anxiety in adult cancer patients. Quality assessment was undertaken using the Newcastle-Ottawa assessment checklists. The quality of each study was further rated using the Agency for Healthcare Research and Quality Standards. Our search yielded 2645 papers, 21 of these were eligible for inclusion. Studies were heterogenous in terms of their characteristics, risk factors and outcomes measured. A total of 32 risk factors were associated with depression and anxiety. We clustered these risk factors into four domains using an expanded biopsychosocial model of health: cancer-specific, biological, psychological and social risk factors. The cancer-specific risk factors domain was associated with the diagnosis, treatment and survivorship phases. Multifactorial risk factors are associated with the onset of depression and anxiety in cancer patients. These risk factors vary across cancer journey and depend on factors such as type of cancer and individual profile of the patients. Our findings have potential applications for risk stratification in primary care and highlight the need for a personalised approach to psychological care provision, as part of cancer care.

## Introduction

Cancer is one of the leading causes of morbidity and mortality globally [1,2]. In the United Kingdom, cancer is the main cause of death, and its prevalence and incidence are expected to increase by over 40% by the next decade [3]. Cancer is not just a physical disease, it is also frequently associated with a psychological burden.

Individuals at different phases of their ‘cancer journey’ exhibit higher psychological symptom levels than the general population [4]. Depression and anxiety specifically are common comorbidities in cancer patients [5,6]. For instance, up to 25% of cancer patients experience depression at some point following diagnosis [7]. Due to similarities between some cancer and depression and anxiety symptoms, such as fatigue [8], such percentages are likely to be underestimates. Cancer patients with comorbid depression or anxiety also have more severe physical and psychological symptoms, and have a poorer prognosis [4,7]. Depression affects cancer survival in many ways as it increases the risk for adverse mental health outcomes such as suicide and Quality of Life (QoL), for instance [8,9]. Despite the known burden of depression and anxiety on cancer patients, co-morbid mental health conditions are often undiagnosed and poorly treated during cancer care [6,8].

Several studies have reported associations with risk factors between different types of cancer and the onset of clinical depression/anxiety [5]. An initial analysis of these risk factors shows a broad categorisation into biopsychosocial and sociodemographic risk factors. Biopsychosocial risk factors include symptoms as a result of cancer or its treatment, social support and psychological factors like fear, distress or pre-existing mental health conditions [5,10,11]. Sociodemographic risk factors include characteristics such as age, sex, marital status and income level [5,8]. Systematic reviews that have examined risk factors associated with depression and anxiety have mainly done so focusing on a specific phase of the cancer journey. For instance, Carreira et al.’s systematic review highlighted risk factors for depression in the breast cancer survivorship phase, after completion of initial curative treatment [12]. Watts et al.’s systematic review and meta-analysis describe these during prostate cancer treatment [9]. However, there is evidence to suggest that the impact of depression/anxiety may be higher at certain phases e.g., during diagnosis and pre/post treatment phases [10]. To our knowledge, no systematic review or single study has synthesised associations between risk factors and mental health outcomes across different phases of the cancer journey.

Currently, cancer patients’ mental health symptoms are more likely to be identified in countries with primary care health services and where primary care plays a crucial role in mental health care [13,14]. For instance, 90% of mental health consultations happen in primary care settings in the UK [13], but many mental health cases in cancer patients and general population can be missed in primary care [15]. Due to the complexity of cancer care, primary care is theoretically well placed for the provision of holistic services in cancer control through navigation through the referral pathway to aid diagnosis and follow-on care, aiding smooth transition across different care settings, provision of community based supportive care during and post treatment, and the overall provision of person-centred care [16,17]. In 2015, the *Lancet Oncology* Primary Care Commission published a report titled “The Expanding Role of Primary Care in Cancer Control” [18]. This report called for an integrated care approach to break the boundary between primary and secondary care, argued for the provision of cancer services in community settings and highlighted the significance of electronic health records in risk assessment and decision making [18].

Comprehensive, longitudinal data is collected on all UK patients in primary care in the form of electronic health records (EHRs) which potentially makes identification of patients at risk of a condition such as a depressive or anxiety disorder much easier, and EHRs systems include a number of risk calculators (e.g., Qrisk) [19,20]. To facilitate and improve the identification of cancer patients with mental health problems using risk calculators more proactively in primary care, we aimed to identify the main mental health risk factors for primary care cancer populations. Our systematic review aimed to 1] understand and synthesise the risk factors associated with depression and anxiety across different cancer types 2] assess whether risk factors would vary across the different phases of the cancer journey: diagnosis, treatment, and survivorship. By focusing on the different types and phases of the cancer journey, our review aimed to identify commonalities and differences in risk factors across cancer populations. We also focused our review on primary and community care settings as this is the likely setting, for most patients, where longer term care is provided, and where most patients will initially seek support for mental health symptoms.

An understanding of the risk factors associated with the onset of depression and anxiety in cancer patients can be used to identify those cancer patients at higher risk of these mental health conditions. This can further inform risk stratification strategies in primary care EHR platforms for early identification and mental health support in cancer patients, and potentially enable early preventative or health promotion interventions personalised for patients in different cancer phases. Preventative interventions informed by risk stratification strategies have the potential to lessen the burden of cancer and mental ill-health on patients and the already stretched health care system.

## Methods

We followed the Preferred Reporting Items for Systematic Review and Meta-Analysis (PRISMA) checklist S1 File and pre-registered a protocol following the PRISMA-P guidelines [21,22] on the PROSPERO international prospective register of systematic reviews (CRD42021283249). The results are also reported in accordance with the Synthesis without meta-analysis checklist guidance (SWiM) in systematic reviews [23] S2 File. The SWiM checklist is a nine-item checklist recently developed to provide a standardised reporting structure for the synthesis of reviews findings, where it is not possible to undertake a meta-analysis [23].

We made the following changes to the protocol after registration:

- Participants: We made pre-existing mental health conditions an exclusion criterion to mitigate confounding effects.
- Outcomes: We streamlined the systematic review outcomes to focus specifically on depression and anxiety, to make our review more manageable. We still included a range of mental health outcomes in our search strategy because the most marginalised populations are least likely to have a clinical diagnosis for depression or anxiety. Our review is now being published under a revised title.
- Context: We clarified that our systematic review would focus on studies recruiting participants from primary care or community settings only.

### Search strategy

We performed an electronic search between April and August 2022 across four databases: PubMed, PsychInfo, Scopus, and EThOS. Search terms for cancer were paired with search terms for mental health outcomes and risk factors using Boolean operators. Full search strings for each database can be found in S1 Table.

### Inclusion criteria

- Participants: Studies involving human adults (>18 years) with cancer, including patients who had just been diagnosed, through to survivorship, and measured anxiety and/or depression as outcome variables. Participants with any type of cancer were included in the review.
- Exposure: Any factor that was examined in relation to risk of anxiety and depression was included.
- Outcomes: Studies were eligible for inclusion if they measured anxiety and/or depression via diagnosis codes in patient medical records, structured clinical diagnostic interviews, screening questionnaires, or by patient self-report. We included a range of measurement for mental health outcomes in our search string because the most marginalised populations are least likely to have a clinician/researcher confirmed diagnosis.
- Setting: Since this review focused on primary care and community settings, we included research cohorts recruited through primary care and community organisations. We also planned to include research cohorts recruited through primary care databases, however we did not find any eligible records for this criterion.
- Study design: Studies that reported original empirical findings and used methods including observational studies, case-control studies, cross-sectional studies, and cohort studies were included.

### Exclusion criteria

- Participants: Since we aimed to synthesise the results by group to determine risk factors for different types of cancer, studies that did not identify risk factors by individual cancer cohorts were excluded. Papers including adult patients who had a pre-existing mental health diagnosis or a history of any type of cancer prior to their current cancer diagnosis were excluded to mitigate confounding effects. Papers were also excluded if they only assessed the mental health and well-being of family members of cancer patients, such as parents of a child with a cancer diagnosis, or those at risk of cancer.
- Outcomes: Papers measuring other outcomes in isolation (i.e., without anxiety and depression), such as neurocognitive dysfunction, fatigue, fear of recurrence, QoL, sleep disturbance, or sexual disfunction were excluded. However, these variables may have acted as risk factors.
- Setting: Studies that recruited participants through settings where they would not be monitored by primary care, such as in-patient cancer settings, hospitals, or via cancer registers were excluded.
- Study design: We excluded papers that measured incidence of mental health issues in cancer patients compared with healthy controls and incidence of mental health outcomes predicting later cancer diagnosis. Literature reviews or systematic reviews were excluded as they did not comprise original empirical findings. Randomised Control Trials (RCTs), or data derived from baseline RCT data, were excluded as they measure the impact of an intervention on mental health outcomes, rather than assessing risk factors for poor mental health.
- Inclusion was also limited to papers written in English as expert translation was unavailable to us. We only included papers published on or before 31^st^ December 2021.

### Study screening and selection

All references identified via database searching were collected in Rayyan reviewing software [24]. Rayyan was used to conduct title and abstract screening. Duplicates were first removed manually after each database search and in Rayyan after the records were uploaded (n = 68). Duplicates were automatically flagged by Rayyan and then reviewed manually by one of the authors (DG). DG reviewed 100% of the titles and abstracts and another author (GG) independently reviewed 25%. Any disagreements were discussed until 100% consensus was reached. Author DI reviewed 100% of the full-texts and DG independently reviewed 25%. Conflicts were again either discussed or resolved by a third reviewer (EF) until 100% consensus was reached on the final set of papers.

#### Data extraction

Two authors (DI and DG) systematically extracted the following information from the included articles into an Excel spreadsheet: author names, publication date, country, study design, participant details (number, sex, age), cancer type, setting, mental health outcome and measurement, prevalence of depression and/or anxiety, risk factors assessed, and main outcomes.

#### Quality assessment

We used the Newcastle-Ottawa Quality Assessment Scale for non-randomized studies to assess the quality of cohort and case control studies [25]. For the cross-sectional studies, we used the modified Newcastle-Ottawa Scale by Herzog et al. [26]. The scales comprise three domains: selection of cases, comparability of cases and measurement of outcome. Items across these domains are given a star rating from 0 (no star) to a maximum of 2 stars per item, with the total number of stars adding up to 10. DI assessed the quality of all the included papers and DG independently reviewed 25%. The inter-rater agreement for the quality assessment was 85.7%. Any conflicts were reviewed by author EF and resolved through discussion.

### Data synthesis

Due to the heterogeneity of the study characteristics and exposures, a meta-analysis of the extracted data was not feasible. Thus, we conducted a narrative synthesis guided by Popay et al.’s four-step narrative synthesis framework [27]. The four non-chronological steps recommended by Popay et al. [27] involve development of theory, preliminary analysis, exploring relations and critical assessment. The preliminary analysis was presented descriptively using tables and visual mapping. We then explored relationships between and among different studies and categorised the risk factors into cancer-specific, biological, psychological and social. Although we did not seek to develop a theory in our study, we adapted the bio-psycho-social model to map the risk factors into the four categories. Although some studies have previously categorised risk factors like age and sex as sociodemographic variables [5,8], in our adapted bio-psycho-social model, we categorised age and sex as biological risk factors. We used the SWiM guidance [23] for the critical assessment phase of the narrative synthesis.

The synthesis identified all possible risk factors assessed in the final set of papers in relation with depression and anxiety. We mapped the risk factors showing significant association with anxiety and/or depression and those with no association with anxiety and/or depression from the studies. We combined findings from all study designs (cross-sectional and longitudinal) in our descriptive analysis and highlighted differences in findings between the different study designs where appropriate.

## Results

### Results of the database search

**Fig 1:**
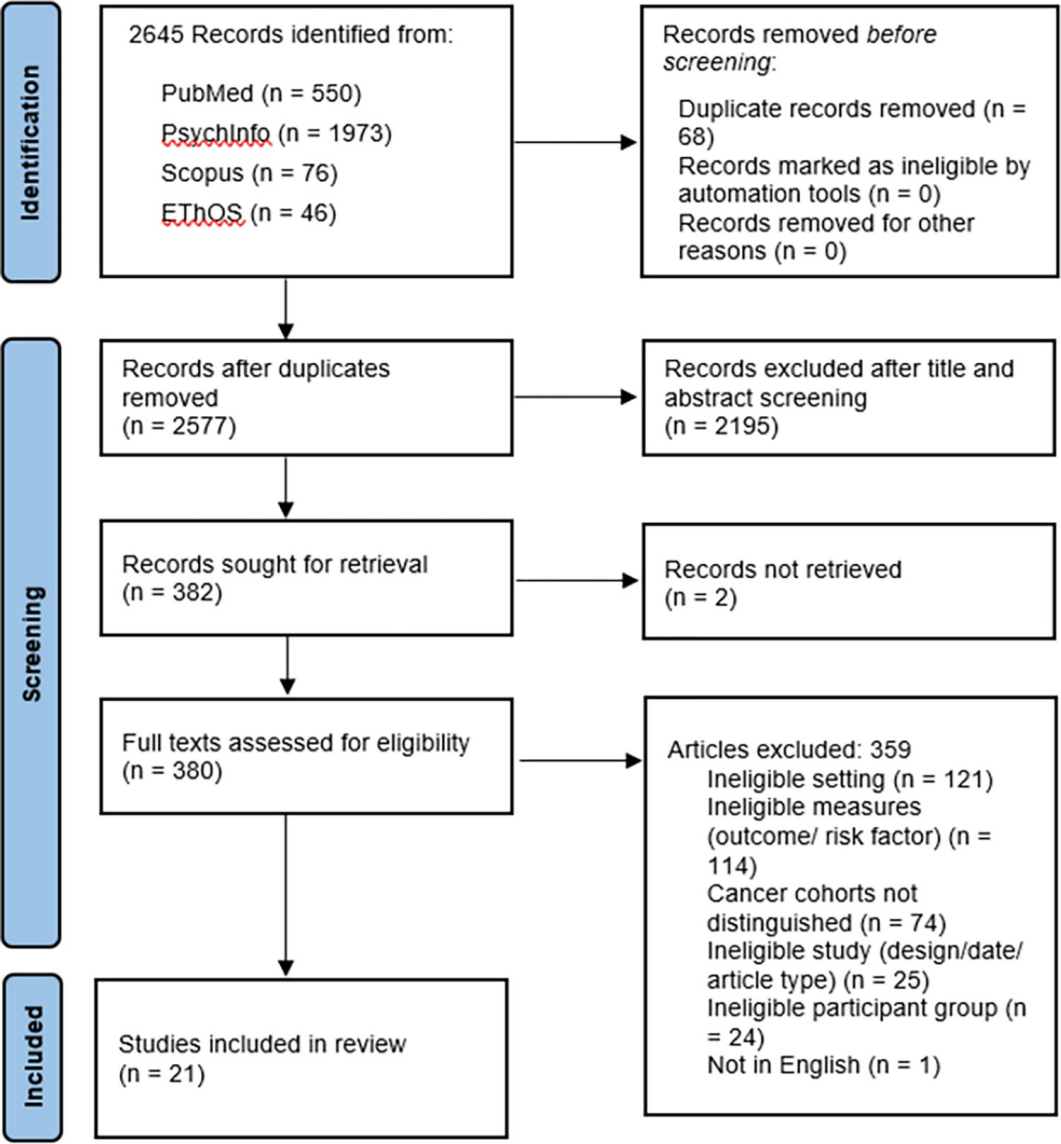
PRISMA flowchart for study selection.

We identified a total of 2645 papers from the four databases searched. 68 duplicates were removed, leaving 2577 for screening. Of these, we excluded 2195 during the title and abstract screening, and a further 359 during the full text screening. We could not retrieve two full texts for screening. This resulted in a final set of 21 papers included in the narrative synthesis Fig 1.

### Quality assessment results

The results of the quality assessment can be found in S2 Table. Majority of the cross-sectional studies (80%) scored between 7 to 9 stars of the Newcastle-Ottawa scale. The lowest score for the cross-sectional studies was 6 stars.

The longitudinal studies generally had lower scores than the cross-sectional, with the highest score of 8 stars. 50% of the longitudinal studies scored 7 to 8 stars. None of the longitudinal studies was rated for assessment of outcome as they all used self-reported tools. The lowest score for the longitudinal studies was 4 stars, and this was predominantly due to a lack of comparability in the study design and analysis. One of the longitudinal studies ascertained that one participant (n = 76) had received a diagnosis of depressive disorder during baseline data collection but had not received antidepressant treatment [28]. We did not exclude the study as we considered that the risk of bias from including the one participant would be insignificant. We further rated the quality of each study as good, fair or poor using the Agency for Healthcare Research and Quality Standards S2 Table. We converted the Newcastle-Ottawa score for each study into AHRQ rating using existing threshold defined by other studies [29]. 16 studies were rated good, while only one study was rated as poor.

### Study characteristics

The studies included in this review were heterogenous in terms of study design, location, study population, setting, cancer types, phase in the cancer journey, risk factors measured, type of measure used and how they were administered as summarised in Table 1. The studies assessed risk factors using a mix of self-report questionnaires and validated scales. The outcomes were also measured using different types of depression/anxiety scales.

**Table 1:** Study characteristics.

Author, Date, Country	Study design	Setting	Participants	Mean age	Cancer type	Outcome & type of measure	Risk factors assessed & type of measure	How outcome & risk factors were measured
Agarwal et al., 2010, United States [30]	Cross-sectional	Outpatient	Older African American cancer survivors (N = 283) Sex: Female (59.7%), Male (40.3%)	63.5 years	Breast cancer: 29.7% Lung cancer: 21.6% Prostate cancer: 7.1% Colon cancer: 12.4% Head and neck cancer: 7.8% Others 21.6%	Depression (GDS-SF15^a^)	• Age • Employment status • Yearly income • Living alone • Health insurance status • Cancer and cancer treatment symptoms • Length of time since diagnosis • Type of cancer • Tumour stage • Stage of cancer at diagnosis	• Self-report questionnaire (interviewer administered)
Davis et al., 2014, United States [31]	Cross-sectional	Community-based	African American breast cancer survivors (at least 1 year post active cancer treatment) (N = 155) Sex: Female (100%)	Not provided (mean age at diagnosis = 51.7 years)	Breast cancer	Depression (BDI-II^b^), Anxiety (BAI^c^)	• Triple Negative Breast Cancer (TNBC) • Hormone receptor (ER, PR) status • Basal Metabolic Index • Chronic stress (Chronic Burden Scale) • Cancer genes (BRCA, p53) • Age at diagnosis ≤45 years • Age of first pregnancy ≤30 years • Income level • Education level	• Self-report questionnaire (Interviewer/self-administered)
Grassi et al., 2010, Italy [32]	Cross-sectional	Outpatient	Cancer patients with diagnosis within 6 months (N = 145) Sex: Female (100%)	55.87 years	Breast cancer	Depression (HAD-D^d^)	• Serotonin transporter (5-HTTLPR) polymorphism • Stressful life events (Life Events Scale) • Type D (personality) (Type-D Scale) • Social support (Multidimensional Scale of Perceived Social Support) • Coping	• Clinical semi-structured interview • Self-report questionnaire • Blood sampling
Hamilton et al., 2013, United States [33]	Cross-sectional	Outpatient	African American cancer patients (N = 77) Sex: Female (66%), Male (34%) 78% were in treatment	Not provided	Breast cancer: 42% Colorectal cancer: 6% Lung cancer: 19% Hematologic cancer: 9% Prostate cancer: 4% Others: 19%	Depression (GDS-SF15 ^a^)	• Religiosity (The Organized Religious Involvement Subscale) • Emotional support (Ways of Helping Questionnaire (WHQ)- Others There for Me subscale) • Collectivism (The Collectivism scale) • Stigma (Adapted stigma scale)	• Self-report questionnaire (interviewer administered)
Hoang et al., 2020, United States [34]	Cross-sectional	Community-based	Immigrant Chinese American breast cancer survivors (N = 110) Sex: Female (100%)	58.43 years	Breast cancer	Depression and Anxiety (BSI^e^)	• Cancer concerns (Profile of Concerns About Breast Cancer Scale) • Seeking social support • Coping (Revised Version of the Ways of Coping Inventory)	• Self-report questionnaire (self-administered)
Jarzemski et al., 2019, Poland [35]	Cross-sectional	Outpatient	Caucasian patients receiving treatment (N = 100) Sex: Male (100%)	Not provided	Prostate cancer	Depression and Anxiety (HADS^d^)	• Treatment modality	• Self-report questionnaire • Review of medical record
Kuswanto et al., 2020, Australia [36]	Cross-sectional	Community-based	Mothers who were breast cancer survivors (N = 91) Sex: Female (100%)	50.87 years	Breast cancer	Depression, Anxiety and Stress Scale (DASS-21^f^)	• Parenting efficacy (Cancer-Related Parenting Self Efficacy (CaPSE)) • Posttraumatic growth (Posttraumatic Growth Inventory Short Form) • Fear of cancer recurrence (Concerns About Recurrence Scale)	• Self-report questionnaire (self-administered)
Mikoshiba et al., 2013, Japan [38]	Cross-sectional	Outpatient	Hepatocellular cancer survivors, 1 year+ curative treatment (N = 127) Sex: Female (36.2%), Male (63.7%)	69 years	Hepatocellular cancer	Depression (Japanese version of CES-D^g^)	• Karnofsky performance status • Liver function • Employment status • Co-habitation status	• Self-report questionnaire (self-administered) • Review of medical records
Lu et al., 2015, United States [37]	Cross-sectional	Community-based	Chinese American breast cancer survivors within 5 years after diagnosis (N = 118) Sex: Female (100%)	54.65 years	Breast cancer	Depression (BSI^e^)	• Ambivalence over emotional expression (Ambivalence over Emotional Expression Questionnaire)	• Self-report questionnaire
Popoola & Adewuya, 2012, Nigeria [39]	Cross-sectional	Outpatient	Women with breast cancer, at least 3 months post diagnosis (N = 124) Sex: Female (100%)	Not provided	Breast cancer	Depression (MINI^h^)	• Stage of cancer • Length of diagnosis • Treatment expenditure • Marital status • Age • Education level • Employment status • Income level • Smoking • Drinking • Treatment expenditure • Perceived social support • Breast cancer family history	• Diagnostic interview • Self-report questionnaire
Przezdziecki et al., 2013, Australia [40]	Cross-sectional	Community-based	Breast cancer survivors (N = 279) Sex: Female (100%) Diagnosis: >3 years	53.4 years	Breast cancer	Depression and Anxiety (DASS21^f^)	• Self-compassion (Self Compassion Scale) • Body image (Body Image Scale) • Perceived pressure from others • Comfort with weight	• Self-report questionnaire (self-administered)
Rice et al., 2018, Canada [41]	Cross-sectional	Community-based	Prostate cancer patients (N = 100) Sex: Male (100%)	64.8 years	Prostate cancer	Depression (PHQ-9^i^/MDRSS-22^j^)	• Co-morbidity	• Self-report questionnaire (self-administered)
Su et al., 2017, Taiwan [42]	Cross-sectional	General hospital	Breast cancer patients (N = 300) Sex: Female (100%)	48.16 years	Breast cancer	Depression (Chinese version of MINI^h^)	• Family support (Family Adaptability, Partnership, Growth, Affection, and Resolve Score) • Hormone therapy • Radiotherapy • Pain severity (Visual Analog Scale) • Age • Insomnia • Psychiatric family history • Time since diagnosis	• Review of medical records • Self-report questionnaire (researcher administered) • Psychiatric diagnostic interview
Tsai & Lu, 2019, United States [43]	Cross-sectional	Community-based	Foreign-born Chinese breast cancer survivors living in the United States (N = 112) Sex: Female (100%)	54.54 years	Breast cancer	Depression (BSI^e^)	• Ambivalence over emotional experience • Intrusive thoughts (intrusion subscale of the Impact of Event Scale)	• Self-report questionnaire (self-administered)
Tung et al., 2018, Taiwan [44]	Cross-sectional	General hospital	Female-specific cancer patients who had undergone treatment for more than 6 months (N = 220) Sex: Female (100%)	Not provided	Breast cancer: 25.9% Uterine cancer: 46.4% Ovarian/Vulvar cancer: 27.7%	Depressive symptoms (CES-D^g^)	• Cancer related symptom distress (symptom distress scale) • Cancer related post-traumatic stress symptoms (Davidson Trauma Scale) • Education level • Occupation status • Cancer stage • Cancer recurrence • Time from diagnosis • Treatment status	• Self-report questionnaire (interviewer administered)
Bright & Stanton, 2018, United States [45]	Longitudinal	Community oncology breast clinic	Women with breast cancer receiving their 1^st^ endocrine therapy prescription (N = 130) Sex: Female (100%)	54.2 years	Breast cancer	Depression (CES-D^g^)	• Social support (Interpersonal Support Evaluation List) • Coping (COPE and Emotional Approach Coping)	• Interview • Interviewer administered questionnaire • Review of medical records
Enns et al., 2013, United States [46]	Longitudinal	Outpatient	Newly diagnosed cancer patients (N = 480) Sex: Female (45%), Male (54.8%)	60.4 years	Gastrointestinal cancer: 24% Prostate cancer: 21.9% Gynaecological cancer: 11.3% Skin cancer: 10.8% Head and neck cancer: 8.3% Others: 23.7%	Depression and anxiety (PSSCAN^k^)	• Age • Sex • Cancer type • Cancer treatment modality	• Self-report questionnaire (self-administered) • Telephone interview
Hsiao et al., 2013, Taiwan [28]	Longitudinal	Outpatient	Breast cancer survivors who received surgical treatments at least 1.99 years prior to study (N = 76) Sex: Female (100%)	50.8 years	Breast cancer	Depression (BDI-II^b^)	• Meaning in life (Meaning in Life questionnaire) • Cortisol responses (saliva cortisol levels using neutral cotton salivette tubes)	• Self-report questionnaire (self-administered) • Saliva sampling
Iwatani et al., 2013, Japan [47]	Longitudinal	Outpatient	Women attending clinic for breast cancer diagnosis (N = 222) Sex: Female (100%)	46.3 years	Breast cancer	Depression and Anxiety (HADS^d^, Japanese version)	• Tumour stage (core needle biopsy) • Spirituality (Functional Assessment of Chronic Illness Therapy—Spiritual subscale)	• Interviewer administered questionnaire • Histopathologic diagnosis
Lee et al., 2015, United States [48]	Longitudinal	Outpatient	Men initiating ADT treatment (N = 61) Sex: Male (100%)	67 years	Prostate cancer	Depression (CES-D^g^)	• Treatment modality	• Self-report questionnaire (self-administered) • Review of medical record
Neilson et al., 2013, Australia [49]	Longitudinal	Outpatient	HNC patients receiving radiotherapy treatment (N = 101) Sex: Female (15.8%), Male (84.2%)	63 years	HNC	Depression and anxiety (HADS^d^)	• Cancer/treatment-related physical symptoms (Functional Assessment of Chronic Illness Therapy-Head and Neck Version- Additional concerns subscale) • Age • Time since diagnosis • Pain • Treatment modality • Sex • Living alone	• Self-report questionnaire (self-administered)

^a^GDS-SF15: Geriatric Depression Scale Short Form.

^b^BDI-II: Beck Depression Inventory–II.

^c^BAI: Beck Anxiety Index.

^d^HADS: Hospital Anxiety–Depression Scale.

^e^BSI: Brief Symptom Inventory.

^f^DASS21—Depression, Anxiety, and Stress Scale.

^g^CES-D: Center for Epidemiologic Studies Depression Scale.

^h^MINI: Mini International Neuropsychiatric Interview.

^i^PHQ-9: Patient Health Questionnaire-9.

^j^MDRS-22—Male Depression Risk Scale-22.

^k^PSSCAN–The Psychological Screen for Cancer.

Fifteen studies used cross-sectional designs [30–44] and the remaining six were longitudinal [28,45–49]. Cross-sectional and longitudinal studies roughly present the same picture. Nine studies were from the United States [30,31,33,34,37,43,45,46,48], one from Italy [32], three from Taiwan [28,42,44], two from Japan [38,47], one from Poland [35], three from Australia [36,40,49], one from Nigeria [39], and one from Canada [41]. In terms of settings, study participants were recruited from general hospitals [42,44], outpatient areas of secondary or tertiary hospitals [28,30,32,33,35,38,39,46–49], and community-based groups or organisations [31,34,36,37,40,41,43]. One study recruited from a community oncology clinic [45], this was included as such clinics follow the ethos of primary care.

The majority of studies focused on risk factors in relation to breast cancer [28,30–34,36,37,39,40,42–45,47], followed by prostate cancer [30,35,41,46,48]. Two of the 21 studies included participants at diagnosis [46,47], nine included participants receiving active treatment [32,33,35,39,41,42,45,48,49], and the remaining 10 comprised participants in the cancer survivorship phase, post active treatment [28,30,31,34,36–38,40,43,44]. One longitudinal study recruited newly diagnosed participants and assessed risk factors across the diagnosis and treatment phases [46].

Depression was the most common outcome, measured by 13 studies [28,30,32,33,37–39,41–45,48].The other eight studies combined depression and anxiety [31,34–36,40,46,47,49], and no study focused on anxiety alone. Table 2 presents an overview of all risk factors associated with an increase in depression or anxiety in patients living with cancer for all the included studies.

**Table 2:** Risk factors associated with depression and anxiety.

Risk factors	Depression only		Anxiety only		Depression and anxiety	
	Number of studies	Total sample size	Number of studies	Total sample size	Number of studies	Total sample size
***Biological risk factors***						
Younger age (<65 years)	1	283	2	581		
Co-morbidity	1	100				
Poor Karnofsky performance status	1	127				
Poor liver function	1	127				
Type D personality	1	145				
Increased cortisol levels at 2100h	1	76				
Higher Basal Metabolic Index			1	155		
***Cancer-specific risk factors***						
Higher level of cancer/treatment related symptoms*	2	503			1	101
Treatment modality	2	361			2	580
Triple Negative Best Cancer (TNBC)					1	155
Advanced stage of cancer	1	129			1	222
Type of cancer			1	480		
Severe pain	1	300				
***Psychological risk factors***						
Low support	3	336			1	110
Lower presence of meaning in life	1	76				
Intrusive thoughts	1	112				
Greater body image disturbance					1	279
Lower self-compassion					1	279
Greater perceived pressure from others					1	279
Less comfort with weight					1	279
Maladaptive coping	1	145				
Higher ambivalence over emotional expression	2	230				
Fear of cancer recurrence					1	91
Poor parenting efficacy					1	91
High cancer concerns					1	110
Higher chronic stress/stressful life events	1	145			1	155
***Social factors***						
Living alone/Unmarried	3	539				
Low religiosity/spirituality	1	77				
High collectivism	1	77				
Lack of health insurance status	1	283				
Unemployment	2	410				
Lower education level	1	220				

*Including cancer related symptom distress, cancer related post-traumatic stress symptoms.

### Risk factors

**Fig 2:**
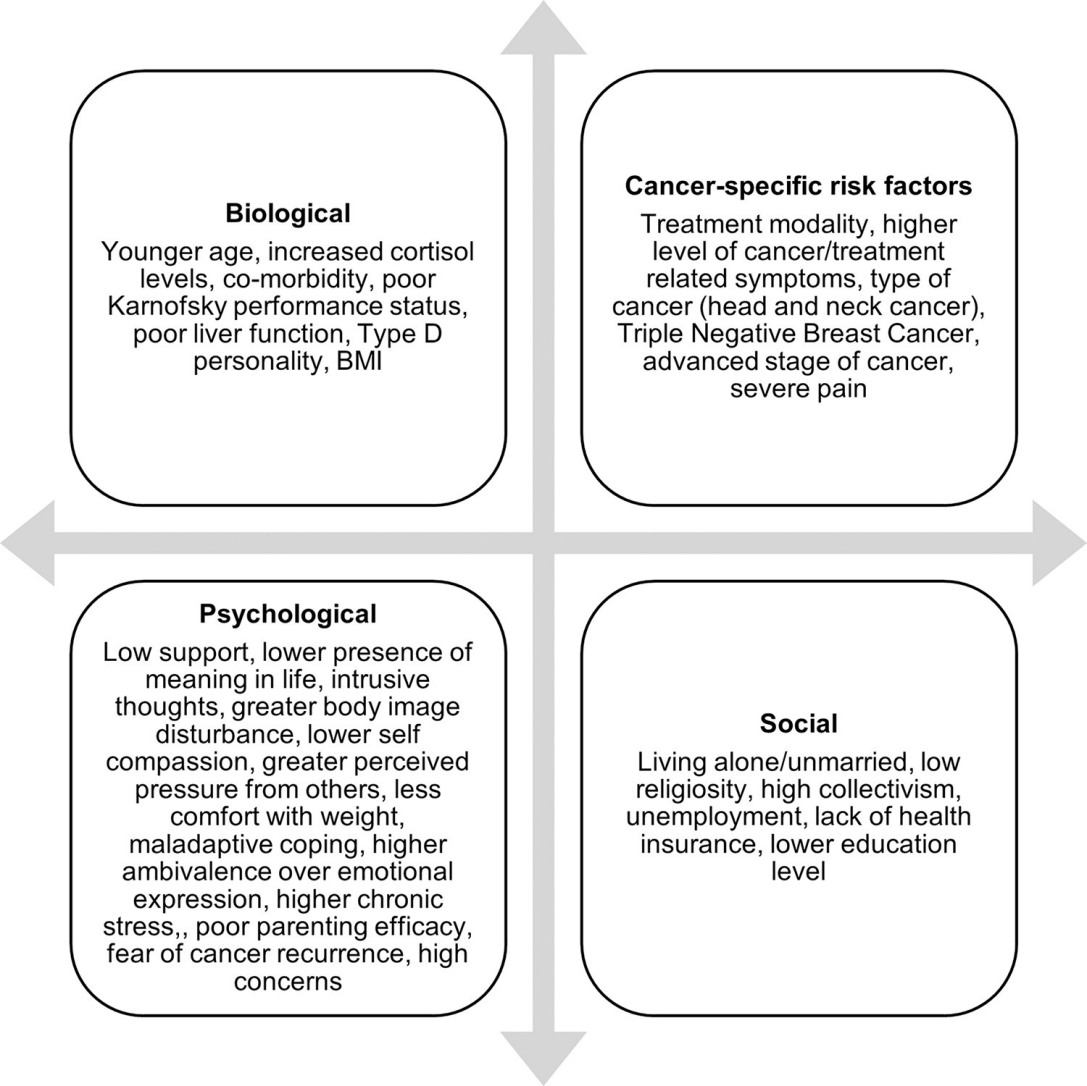
Adapted biopsychosocial model of risk factors for depression and anxiety in cancer patients.

52 risk factors were assessed across the 21 studies reviewed, and 32 of these were associated with depression and anxiety. These risk factors are clustered into cancer-specific, biological, psychological and social domains Fig 2.

#### Biological risk factors

From our synthesis, 10 studies assessed 10 biological risk factors in relation with depression and anxiety [28,30–32,38,39,41,42,46,49], eight of these identified seven biological risk factors associated with depression/anxiety [28,30–32,38,41,46,49]. Six studies assessed age as a possible risk factor for depression and anxiety [30,31,39,42,46,49], only three showed an associated between age and depression/anxiety. Younger age (<65 years) was associated with increased risk of depression among African American cancer survivors [30]. Younger age was also associated with anxiety but not depression in head and neck patients receiving radiotherapy [49], and among newly diagnosed cancer patients [46], although the age in years was not specified. Age at cancer diagnosis ≤45 years and age of first pregnancy ≤30 years were not associated with depression or anxiety in any of the studies. Two studies explored sex as a possible risk factor for depression and anxiety, but none showed an t association [46,49].

Other biological risk factors associated with increased risk of depression included: increased cortisol levels at 2100 hours in breast cancer survivors [28], co-morbidity, specifically cardiovascular diseases and arthritis in prostate cancer patients [41], poor Karnofsky performance status (score less than 80) and poor liver function in hepatocellular cancer survivors [38]. Higher Basal Metabolic Index was associated with anxiety, but not depression in African American breast cancer survivors [31].

#### Cancer-specific risk factors

From our synthesis, 11 studies identified eight possible cancer-specific risk factors for depression and anxiety [30,31,33,35,39,42,44,46–49], 10 of these identified six cancer-specific risk factors associated with increased depression/anxiety [30,31,35,39,42,44,46–49]. Cancer treatment modality showed associations with depression and anxiety in four studies [35,42,46,48]. Receiving chemotherapy as treatment for cancer was associated with anxiety and depression in Enn et al.’s multi cancer study [46]. Adjuvant therapy, specifically radiotherapy and androgen-deprivation therapy (ADT) was associated with the presence of depression and anxiety in men undergoing radical prostatectomy [35]. Similarly, administering ADT to prostate cancer patients was associated with depression [48]. Lastly, hormone therapy and radiotherapy were identified as risk factors for depression in breast cancer patients [42].

Symptoms related to cancer (including cancer-specific stress, cancer related post-traumatic stress) or cancer treatment was associated with increase in depression in breast cancer survivors [30,44,49] and anxiety in head and neck cancer patients [49].

The type of cancer, specifically head and neck cancer and the aggressiveness of triple negative breast cancer were associated with increased risk of anxiety among newly diagnosed cancer patients [46] and breast cancer survivors [31] respectively. Advanced tumour stage following diagnosis of breast cancer was also associated with depression [39,47] and anxiety [47].

Time since diagnosis was not associated with depression or anxiety in any of the studies.

#### Psychological risk factors

A total of 11 studies assessed 15 psychological risk factors for depression and anxiety [28,31–34,36,37,40,42,43,45], 10 of these studies highlighted 13 risk factors associated with increased depression/anxiety [28,31–34,36,37,40,43,45]. Four studies associated having low support levels (including family support, emotional support and support as a result of quality of the physician-patient interaction) with depression [33,34,39,45]. Perceived less meaning in life and its constant decrease over the 14 months period of a longitudinal study were associated with depression in breast cancer survivors [28]. Being more ambivalent over emotional expression was linked with depression among Chinese American breast cancer survivors [37,43]. In the three studies that explored coping mechanisms [32,34,45], only one found an association between maladaptive coping and higher risk of depression [32]. Chronic stress was associated with depression and anxiety in African American breast cancer survivors [31]. Similarly, stressful life events were linked with depression in breast cancer patients in Italy [32]. Lower self-compassion, less comfort with weight, greater perceived pressure from others and greater body image disturbance were associated with depression and anxiety in breast cancer survivors [40]. Other psychological risk factors associated with depression and anxiety included intrusive thoughts among breast cancer survivors [43], poor parenting efficacy [36], fear of cancer recurrence [36], and high cancer concerns [34].

#### Social risk factors

Eight studies identified nine social risk factors for depression and anxiety for people living with cancer [30,31,33,38,39,44,47,49], six of these highlighted six social risk factors associated with increased depression/anxiety [30,33,38,39,44,49]. The most common social factor, reported in four studies, was the living situation of participants, whether they were married, co-habiting or living alone [30,38,39,49]. Three out of these studies showed significant associations between living alone or being unmarried as risk factors for depression [30,38,39], while living alone was not associated with depression or anxiety in Neilson et al.’s longitudinal study [49]. Low involvement in organised religious activities and collectivism were risk factors associated with depression in older African American cancer patients [32], however spirituality did not have a significant association in Iwatani et al.’s [47].

Socioeconomic status including education, income level and employment status showed contradicting associations with depression and anxiety. Lack of unemployment or losing job as a result of poor health were associated with depression in breast cancer and hepatocellular cancer survivors [30,38]. However, employment status was not associated in Popoola’s study involving breast cancer patients in Nigeria [39]. Having no health insurance [30] and low-income level (<$20,000) were also associated with depression [33]. Davis et al.’s study had a higher threshold for low income (<$50,000) and found no association between income level and depression or anxiety [39]. Although three studies assessed education as potential risk factors for depression and anxiety [31,39,44], only one showed an association between lower education level and increased risk of depression [44].

The risk factors associated with depression and anxiety are presented using an adapted biopsychosocial model Fig 2.

#### Summary of risk factors by outcome

We also present a summary of risk factors for both outcomes in Table 2. We identified 22 risk factors for depression only cutting across all the four domains of our adapted biopsychosocial model, whereas three risk factors were associated with anxiety only. The risk factors associated with anxiety were related to the biological and cancer-specific domains of the adapted biopsychosocial model. We also identified 13 risk factors reported in the studies that measured both depression and anxiety.

We further present the risk factors associated with depression and anxiety in the different cancer types identified from our review (Table 3).

**Table 3:** Risk factors presented by cancer types.

Type of cancer	Higher risk of Depression	Higher risk of Anxiety
Breast cancer	Advanced stage of cancer Type D personality, hormone therapy, radiotherapy, severe pain, low support, maladaptive coping, living alone/unmarried TNBC, chronic stress, low support, high cancer concerns, poor parenting efficacy, fear of cancer recurrence, intrusive thoughts, high ambivalence over expression, greater body image disturbance, greater perceived pressure from others, less comfort with weight, low self-compassion	Advanced stage of cancer Basal mass index, cortisol levels, TNBC, low support, chronic stress, high cancer concerns, lower presence of meaning in life, greater body image disturbance, greater perceived pressure from others, less comfort with weight, low self-compassion
Prostate cancer	ADT, radiotherapy, co-morbidity	Adjuvant therapy
Head and neck cancer	Higher cancer/treatment related symptoms	Younger age, Higher cancer/treatment related symptoms
Hepatocellular cancer	Poor Karnofsky performance status, poor liver function, unemployment, living alone/unmarried	
Female-specific cancer (Breast cancer, uterine cancer, ovarian/vulvar cancer)	Higher cancer/treatment related symptoms, low education level	
Multi cancer	Chemotherapy, low support, low religiosity, collectivism, cancer/treatment related symptoms, unemployment, living alone/unmarried, age, lack of health insurance	Type of cancer (Head and neck cancer), chemotherapy, younger age

### Risk factors across the cancer journey

**Fig 3:**
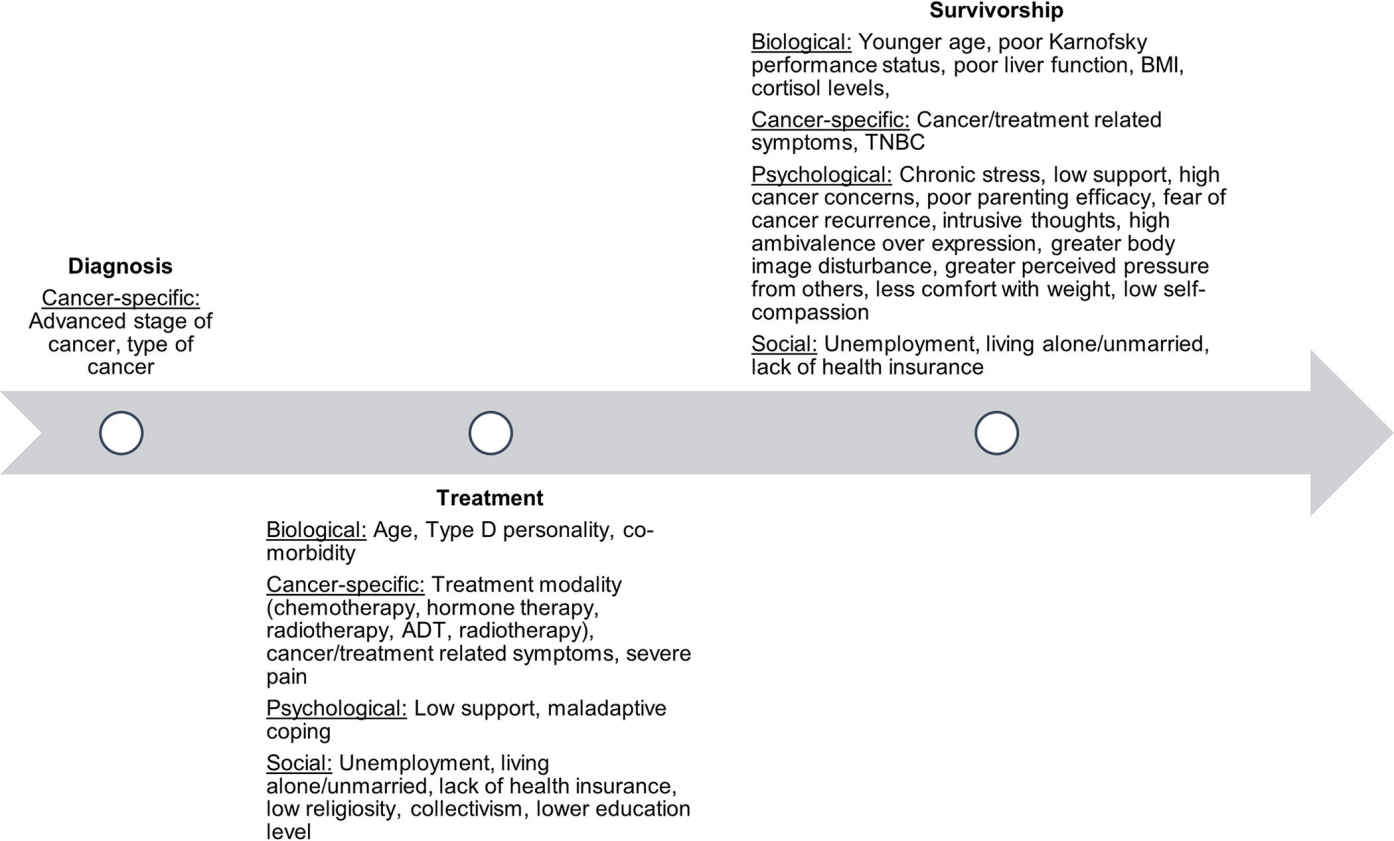
Summary of risk factors across the diagnosis, treatment and survivorship phases.

The risk factors associated with depression and anxiety across the diagnosis, treatment and survivorship phases of cancer are outlined in Fig 3. Although the broad domain of cancer-specific risk factors was consistent across the three phases of the cancer journey, no individual risk factor was common to all three phases. Younger age, higher cancer/treatment related symptoms, low support, unemployment, lack of health insurance, and living alone/unmarried were associated with higher risk of depression and anxiety during the treatment and survivorship phases.

## Discussion

Depression and anxiety are recognised as important comorbid conditions associated with cancer [7]. This systematic review and narrative synthesis have enabled us to synthesise risk factors for depression and anxiety for different cancer types, and across the diagnosis, treatment and survivorship phases of cancer. Through this review, we have been able to identify the independent risk factors for different cancer populations, as well as map out commonalities and differences in risk factors for these populations. We identified that the risk factors can be summarised using a biopsychosocial model. Such models are typically conceptualised as encompassing biological, psychological, social domains [7,10]. To conceptualise risk factors for depression and anxiety in this current study, we included a fourth domain, cancer-specific factors, to differentiate between general biological risk factors and those relating specifically to cancer.

Psychological risk factors were the most prominent category of risk factors associated with depression and anxiety despite the exclusion of pre-existing mental health conditions in our review. This emphasises that other psychological factors are crucial in the mental health and well-being of cancer patients. Low support from families, friends and the healthcare system was a common risk factor at both the treatment and survivorship phases of breast cancer whereas it was only found to be a risk factor for anxiety at the survivorship phase. Other studies have reported low social support from family, friends and social networks as a risk factor in patients with a cancer diagnosis and those receiving treatment [5,50]. Other psychological risk factors, particularly those regarding appearance and views of the self, such as body image, perception of meaning in life and self-compassion, were associated with depression and anxiety at the survivorship phase of breast cancer. This finding is consistent with a vast body of literature that has shown that the aftermath of breast cancer treatment results in physical changes which have huge psychological impacts on survivors [12,51].

Contrary to views that risk factors for depression and anxiety are more related to individual factors such as younger age rather than to cancer- and treatment-specific symptoms [52], our review showed that the domain of cancer-specific risk factors was consistent across the three phases of the cancer journey. We also identified cancer symptoms and treatment symptoms (e.g. pain, cancer specific distress) as risk factors for depression and anxiety in this review, similar to other study findings [5,11]. Specifically, we observed that treatment modality, type of cancer, advanced cancer, TNBC, and symptoms associated with cancer, or its treatment, were prominent cancer-specific factors. Consistent with a recent systematic review, we also found that head and neck cancer may be a particular independent risk factor for depression and anxiety [5]. The reason for this may be because this type of cancer is aggressive and generally has a low survival rate, and the treatment can cause side effects which are unpleasant to live with like xerostomia, changes to tongue and mouth, and visual differences of the face [53].

In contrast to Riedl’s systematic review [5], treatment modality was also associated with depression. We found the use of ADT and radiotherapy as main or adjuvant therapy as risk factors in prostate cancer patients, hormone therapy and radiotherapy as risk factors for depression in breast cancer patients, while chemotherapy, radiotherapy and hormone therapy were identified as risk factors for other cancer types. The stage of tumour at diagnosis, dependent on the type of cancer, may inform the treatment modality. Hence, it is important to understand the interactions between these three factors when making decisions about mental health care planning for a cancer patient.

Biological factors, specifically a younger age, was the most common risk factor associated with depression and anxiety in our review. This factor has previously been associated with general mental health outcomes in cancer patients. For instance, a 5-year observational cohort study in the UK identified younger age as a risk factor for depression and anxiety in women post breast cancer diagnosis [52]. Recent studies have also shown that younger age is associated with depression and anxiety during cancer treatment [54]. However, there was no consistent observable pattern in the association between age and depression/anxiety across the three phases of the cancer journey or for different cancer types. Therefore, there is a need for further studies to explore how younger age acts as an independent risk factor among different cancer cohorts.

Since social risk factors, specifically measures of socioeconomic status (SES) such as employment status, income level and education level are important determinants of access to health care and overall health outcomes [55], we expected these also to be associated with depression and anxiety for people living with cancer. However, the findings from the collection of studies in our review did not consistently link social factors with depression and/or anxiety in this patient group. For instance, the findings from our review showed discrepancy in employment status. Some studies found unemployment to be associated with depression and anxiety during survivorship while others did not [30,39]. We also expected low income to be closely associated with treatment phase due to the possible impact of economic situation on access to cancer care [56], but we identified no significant associations. The reason for this may be due to the discrepancy in the threshold for low income across the studies included in this review. For instance, Hamilton et al.,’s threshold of US$20,000 was a significant risk factor but Davis et al.’s threshold of US$50,000 was not [31,33]. Therefore, individuals earning lower income or those with greater economic deprivation may be at a higher risk of depression.

Living alone was another social risk factor, which was associated with depression, but not anxiety. This is consistent with other evidence suggesting living alone can be a risk factor for depression [12]. This is unsurprising given the greater likelihood for social isolation, and potential that those living alone have lower social support [57]. We have demonstrated the inevitable complexity of the association between SES and depression/anxiety. Further studies are required to clarify the associations between different markers of SES and depression and anxiety in cancer patients to allow targeted support for those in most need.

Our findings suggest that some biological and cancer-specific risk factors are specific to the type of cancer. For instance, poor Karnofsky performance status and poor liver function was associated with depression in head and neck cancer patients and TNBC associated with breast cancer survivors [31,32].

Lastly, our review provided some evidence for variation in risk factors among different ethnic groups. The finding relating to TNBC suggests that there may be unique risk factors associated with certain ethnic groups, for instance TNBC is more common among women of African [58]. While collectivism has been identified as a protective factor among certain ethnic groups especially those from African and Asian communities [59], our findings show the reverse as high collectivism was associated with depression in cancer patients. Therefore, further studies are required to understand risk factors that may be specific to different population groups.

### Implications for health care research and clinical practice

This study has potential implications for healthcare research and clinical practice. As our review reveals that multiple risk factors are associated with depression and anxiety in cancer patients, there is a need to raise awareness among primary health care providers on the various risk factors, especially social risk factors like socioeconomic status. Better awareness of the various risk factors could enable primary care providers better identify mental ill-health and inform personalised treatment pathways for cancer patients. A more robust understanding of the risk factors at different cancer phases and for different cancer types can also inform risk stratification in cancer care. For example, younger age is a prominent risk factor, as such routine enquiry about depression and anxiety may be especially relevant for younger cancer patients. However, our review has shown that risk factors for depression and anxiety vary by cancer, and potentially by age and socioeconomic status. Therefore, a one-size fits-all risk calculator for mental health problems following cancer diagnosis is unlikely to perform with high accuracy; instead, next steps should examine risk factors for specific cancers.

A good starting point for risk stratification in cancer care would be to develop and trial a risk calculator for depression and anxiety following cancer diagnosis. We recommend conducting this trial with breast cancer diagnosis in the first instance, as this is a common cancer and has the most research available; in addition, there are a number of evidence-based interventions available for supporting mental health in this cancer cohort [60–62]. A proof-of-concept risk calculator for this group of patients would then provide evidence that understanding risk factors for mental ill-health can be valuable to support the allocation of healthcare resources, and support further research into risk factors for depression and anxiety in other cancers. It will be important to involve primary care clinicians, patients, carers and healthcare service commissioners in this research to ensure that where patients with depression and anxiety are identified, evidence-based therapy and support is made available.

Cancer treatment can generally be invasive with prominent side effects which do not necessarily resolve at the end of active treatment. This review demonstrates the potential for certain treatments to also trigger poor mental health outcomes, particularly chemotherapy, radiotherapy, ADT and hormone therapy. Therefore, the treatment of and care for people living with cancer should encompass not only attention to the cancer and the presenting or subsequent physical symptoms but should include psychological care to minimise the risk of depression, anxiety and other forms of mental disorders and to improve quality of life in cancer survivors living with ongoing side-effects of treatment.

### Strengths and limitations

This is the first study to our knowledge that has aimed to synthesise risk factors for depression and anxiety in cancer patients from diagnosis through to survivorship. Risk factors for mental ill-health in cancer patients have so far not been concretely characterised [7]. Our review addresses this gap in understanding by presenting the risk factors that may be unique to different cancer stages: diagnosis, treatment and survivorship. Also, the use of the expanded biopsychosocial model of health enabled us to characterise risk factors in a structured manner and provides a valuable framework for future researchers. Another strength of our review is the use of a narrative synthesis which enabled us to combine cross-sectional and longitudinal study outcomes, overcoming variability in the study designs [27].

We restricted the studies included in this review to those conducted in primary and community care settings, which strengthens the relevance of our findings for primary care provision. However, while we sought to review the identification of risk factors on primary care records or datasets, we did not find any papers that used primary care records that were eligible for inclusion. We did not search through any primary care database in our review. This was a limitation to our search strategy and may have contributed to the lack of identification of relevant papers on primary care records. The exclusion of pre-existing mental health conditions in our review is another limitation as this is likely to be one of the most important risk factors for an EHR calculator.

Most studies were from upper-middle and high-income countries, except from one study conducted in Nigeria, which limits the applicability of our findings to lower-middle and low-income countries. More studies are required in these regions of the world especially as mental health and cancer are two growing health conditions of global significance.

Given the higher prevalence and treatment outcomes of certain types of cancer, it is unsurprising that most studies focus on these (e.g., breast cancer and prostate cancer). For example, 68% of the eligible papers from our review focused on breast cancer. Studies that examined multiple types of cancer also considered breast cancer, except for one. The focus on “popular” cancers may inadvertently lead to neglect for patients with less common cancers. There is a need for more research generally on other cancer types to strengthen the evidence base and ensure adequate mental health care for subset of cancer patients who might be underserved. In addition, the wide focus on all cancer types and phases limited our ability to identify a coherent group of clear risk factors which could be operationalized into a risk score or risk calculator. Further work on this aim would need to proceed on a cancer-by-cancer basis, as results obtained in this review are difficult to interpret and apply in specific clinical contexts.

The studies in our review used various measures to assess the risk factors. This was a strength to our review as it enabled us include different population groups, for instance, marginalised groups who may not have access to formal diagnosis of mental health disorders. However, it might limit the robustness and clinical utility of our findings. The use of diagnostic interviews in primary care studies is not common due to how resource intensive these interviews are to administer, yet the use of self-reported scales has been highlighted to come with limitations like lack of standardisation [12,15,63]. The interpretation and clinical utility of our findings may have some limitations due to the different self-report measures and scales used to assess depression and outcomes, which may or may not meet clinical thresholds.

## Conclusion

Our systematic review and narrative synthesis sought to understand the underlying risk for depression and anxiety among cancer patients at the time of diagnosis, during treatment, and through to survivorship. We focused our review on risk factors identified within primary and community care settings. We demonstrated that the multifactorial risk factors associated with depression and anxiety in cancer patients can be represented through an expanded biopsychosocial model comprising four domains: biological, cancer-specific, psychological and social risk factors. A crucial finding was a divergence in risk factors at different stages of cancer progression and by cancer type. This underscores the need for a personalised approach to psychological care provision as part of cancer care as interventions may depend on the cancer type, population characteristics and phase of cancer care.

## Supporting information

S1 Table Search strategy. (DOCX)

S2 Table Quality assessment results. (DOCX)

S1 File PRISMA checklist. (DOCX)

S2 File SWiM checklist. (DOCX)
