# Supplementary material for: A systematic review of risk factors associated with depression and anxiety in cancer patients
Source: PLoS One. 2024 Mar 29;19(3):e0296892. doi: 10.1371/journal.pone.0296892 (PMC10980245; doi:10.1371/journal.pone.0296892)
Supplement: S1 Table — (DOCX) [file pone.0296892.s001.docx]

**PubMed**

| Search String | (((Cancer OR Tumo* OR Neoplas* OR Carcinoma*) AND (Depress* OR Anxi* OR "anxiety disorder" "sexual dysfunction" OR "neurocognitive disorder" OR "cognitive disorder" OR "sleep wake disorders" OR "trauma disorders" OR "stress disorders" suicide OR somatoform OR "somatoform disorders" OR "bipolar disorder" OR "bipolar and related disorders" OR bipolar OR "obsessive-compulsive disorders" OR "OCD") AND (Risk* OR "Risk factor*")) AND NOT (Child OR Infant OR Childhood OR Adolesce* OR Animals)) |
| --- | --- |
| Limits | English only |

**PsychInfo**

| Search String | (((Cancer OR Tumo* OR Neoplas* OR Carcinoma*) AND (Depress* OR Anxi* OR "anxiety disorder" "sexual dysfunction" OR "neurocognitive disorder" OR "cognitive disorder" OR "sleep wake disorders" OR "trauma disorders" OR "stress disorders" suicide OR somatoform OR "somatoform disorders" OR "bipolar disorder" OR "bipolar and related disorders" OR bipolar OR "obsessive-compulsive disorders" OR "OCD") AND (Risk* OR "Risk factor*")) AND NOT (Child OR Infant OR Childhood OR Adolesce* OR Animals)) |
| --- | --- |
| Limits | Record type: Journal; Methodology: Clinical Case Study, Field Study, Focus Group, Follow up Study, Interview, Longitudinal Study, Nonclinical Case Study, Qualitative Study, Quantitative Study, Treatment Outcome; English only, peer reviewed |

**Scopus**

| Search String | (((Cancer OR Tumo* OR Neoplas* OR Carcinoma*) AND (Depress* OR Anxi* OR "anxiety disorder" "sexual dysfunction" OR "neurocognitive disorder" OR "cognitive disorder" OR "sleep wake disorders" OR "trauma disorders" OR "stress disorders" suicide OR somatoform OR "somatoform disorders" OR "bipolar disorder" OR "bipolar and related disorders" OR bipolar OR "obsessive-compulsive disorders" OR "OCD") AND (Risk* OR "Risk factor*")) AND NOT (Child OR Infant OR Childhood OR Adolesce* OR Animals)) |
| --- | --- |
| Limits | Record type: article, conference paper; English only |

**EThOS**

| Search String | Cancer AND "Mental Health"; Cancer AND Depress*; Cancer AND Anxi* |
| --- | --- |
| Limits | Record type: article, conference paper; English only |
