# Supplementary material for: A systematic review of risk factors associated with depression and anxiety in cancer patients
Source: PLoS One. 2024 Mar 29;19(3):e0296892. doi: 10.1371/journal.pone.0296892 (PMC10980245; doi:10.1371/journal.pone.0296892)
Supplement: S2 Table — (DOCX) [file pone.0296892.s002.docx]

**_Quality assessment results_**

| **_Authors_** | **_Selection_** | | | | **_Comparability_** | **_Outcome_** | | | |  |
| --- | --- | --- | --- | --- | --- | --- | --- | --- | --- | --- |
|  | **_Representativeness of the sample_** | **_Sample size_** | **_Non-respondents_** | **_Ascertainment of the exposure_** | **_Based on study design/analysis_** | **_Assessment of outcome_** | **_Statistical test_** | | **_Total (/10)_** | **_AHRQ rating_** |
| **_Cross-sectional studies_** | | | | | | | | | |  |
| _Agarwal et al._ | _*_ |  | _*_ |  | _**_ | _*_ | _*_ | | _6_ | _Fair_ |
| _Davis et al._ | _*_ | _*_ | _*_ | _*_ |  | _*_ | _*_ | | _6_ | _Fair_ |
| _Grassi et al._ | _*_ |  | _*_ | _**_ | _**_ | _*_ | _*_ | | _8_ | _Good_ |
| _Hamilton et al._ | _*_ |  | _*_ | _**_ | _**_ | _*_ | _*_ | | _8_ | _Good_ |
| _Hoang et al._ | _*_ | _*_ | _*_ | _**_ |  | _*_ | _*_ | | _7_ | _Good_ |
| _Jarzemski et al._ | _*_ | _*_ | _*_ | _**_ | _*_ | _*_ | _*_ | | _8_ | _Good_ |
| _Kuswanto et al._ | _*_ |  | _*_ | _**_ | _**_ | _*_ | _*_ | | _8_ | _Good_ |
| _Lu et al._ | _*_ | _*_ | _*_ | _**_ | _**_ | _*_ | _*_ | | _9_ | _Good_ |
| _Mikoshiba et al._ | _*_ | _*_ | _*_ | _**_ | _**_ | _*_ | _*_ | | _9_ | _Good_ |
| _Popoola & Adewuya_ | _*_ | _*_ | _*_ | _*_ |  | _*_ | _*_ | | _6_ | _Fair_ |
| _Przezdziecki et al._ | _*_ | _*_ |  | _**_ | _**_ | _*_ | _*_ | | _8_ | _Good_ |
| _Rice et al._ | _*_ | _*_ |  | _*_ | _**_ | _*_ | _*_ | | _7_ | _Good_ |
| _Su et al._ | _*_ | _*_ | _*_ | _**_ |  | _*_ | _*_ | | _7_ | _Good_ |
| _Tsai & Lu_ | _*_ | _*_ |  | _**_ | _**_ | _*_ | _*_ | | _7_ | _Good_ |
| _Tung et al._ | _*_ | _*_ | _*_ | _**_ |  | _*_ | _*_ | | _7_ | _Good_ |
| **_Longitudinal studies_** | | | | | | | | | |  |
|  | **_Representativeness of exposed cohorts_** | **_Selection of non-exposed cohorts_** | **_Ascertainment exposure_** | **_Demonstration that outcome not present at start of study_** | **_Based on study design/analysis_** | **_Assessment of outcome_** | **_Length of follow/up >3 months_** | **_Adequacy of follow-up_** |  |  |
| _Bright & Stanton_ | _*_ |  | _*_ |  | _**_ |  | _*_ | _*_ | _6_ | _Fair_ |
| _Enns et al._ | _*_ | _*_ | _*_ |  | _**_ |  | _*_ |  | _6_ | _Good_ |
| _Hsiao et al._ | _*_ | _*_ | _*_ | _*_ | _**_ |  | _*_ | _*_ | _8_ | _Good_ |
| _Iwatani et al._ | _*_ | _*_ | _*_ | _*_ | _*_ |  | _*_ | _*_ | _7_ | _Good_ |
| _Lee et al._ | _*_ | _*_ | _*_ | _*_ | _**_ |  | _*_ | _*_ | _8_ | _Good_ |
| _Neilson et al._ | _*_ | _*_ | _*_ |  |  |  | _*_ |  | _4_ | _Poor_ |
